# Treatment with efavirenz extends survival in a Creutzfeldt-Jakob disease model by regulating brain cholesterol metabolism

**DOI:** 10.1172/jci.insight.190296

**Published:** 2025-06-19

**Authors:** Tahir Ali, Jessica Cashion, Samia Hannaoui, Hanaa Ahmed-Hassan, Hermann Schatzl, Sabine Gilch

**Affiliations:** 1 Calgary Prion Research Unit, Faculty of Veterinary Medicine;; 2 Hotchkiss Brain Institute, Cumming School of Medicine; and; 3 Snyder Institute for Chronic Diseases, University of Calgary, Calgary, Alberta, Canada.

**Keywords:** Infectious disease, Therapeutics, Cholesterol, Neurodegeneration, Prions

## Abstract

Prion diseases are fatal, infectious, and incurable neurodegenerative conditions affecting humans and animals, caused by the misfolding of the cellular prion protein (PrP^C^) into its pathogenic isoform, PrP^Sc^. In humans, sporadic Creutzfeldt-Jakob disease (sCJD) is the most prevalent prion disease. Recently, we demonstrated that treatment with the FDA-approved anti-HIV drug efavirenz (EFV) significantly reduced PrP^Sc^ and extended survival of scrapie prion–infected mice. Among other effects, EFV activates the brain-specific cholesterol-metabolizing enzyme, CYP46A1, which converts cholesterol into 24S-hydroxycholesterol (24S-HC). However, drugs effective against scrapie prions often fail in human prion diseases, and a relation of the antiprion effects of EFV to CYP46A1 activation is not established. Thus, we evaluated EFV treatment in mice overexpressing human PrP^C^ infected with human sCJD prions. Oral, low-dose EFV treatment starting at 30 or 130 days postinfection significantly slowed disease progression and extended their survival. At early clinical stage, we observed reduced PrP^Sc^ accumulation, decreased cholesterol and lipid droplet content, and elevated CYP46A1 and 24S-HC levels in EFV-treated mice. Overexpression of CYP46A1 in prion-infected neuronal cells reduced PrP^Sc^ levels and increased 24S-HC, indicating that antiprion effects of EFV correlate with CYP46A1 activation. These findings highlight EFV as a safe and efficacious therapeutic candidate for human prion diseases.

## Abstract 2

Prion diseases are fatal, untreatable neurodegenerative disease. We demonstrate that treatment with efavirenz extends survival when applied to a mouse model of human prion disease.

## Introduction

Prion diseases, also referred to as transmissible spongiform encephalopathies, represent a group of fatal and incurable neurodegenerative disorders in both humans and animals. Among humans, Creutzfeldt-Jakob disease (CJD) stands out as the main prion disease. CJD exists in various forms: sporadic, familial, and acquired by infection (iatrogenic or variant CJD). Sporadic CJD (sCJD) arises without any known cause and accounts for roughly 85% of human prion diseases. It leads to death within several months upon onset of clinical signs, such as rapidly progressing dementia. Familial CJD stems from inherited mutations in the prion protein (*PRNP*) gene, while iatrogenic and variant CJD are associated with infection from medical procedures and zoonotic transmission of cattle prions, respectively. Other human prion diseases include Kuru, fatal familial insomnia, and Gerstmann-Sträussler-Scheinker syndrome. In animals, prion diseases encompass scrapie in sheep and goats, bovine spongiform encephalopathy in cattle, and chronic wasting disease in cervids (1–12).

Prion diseases are characterized by their prolonged silent incubation period, short clinical stage, and invariably fatal outcome. Histopathologically, they are primarily marked by the accumulation of abnormally aggregated prion protein (PrP^Sc^), which leads to significant brain atrophy associated with spongiosis and gliosis (1–5). The conformational conversion of cellular prion protein (PrP^C^) into its abnormal form (PrP^Sc^) within the central nervous system plays a central role in the pathogenesis of prion diseases. PrP^C^ is highly expressed in the brain and particularly in neurons and astrocytes, modified by up to 2 N-linked glycans, and anchored to cholesterol-rich lipid rafts in the outer leaflet of the plasma membrane via a glycosylphosphatidylinositol anchor. While PrP^C^ predominantly exhibits an α-helical structure, PrP^Sc^ aggregates form a parallel in-register β-sheet structure and are partially resistant to degradation by proteases like proteinase K (PK) (13–17). Cholesterol plays a critical role in the prion conversion process, as treatments that lower cholesterol levels reduce prion propagation, likely through impairment of lipid raft integrity. In prion-infected cells, expression of transcription factors and genes involved in cholesterol biosynthesis is upregulated, contributing to elevated cholesterol levels in neurons and brains of scrapie prion–infected mice, which might favor the conversion of PrP^C^ into PrP^Sc^. In addition, prion infection impairs the ability of the ATP-binding cassette transporter type A1 to facilitate cholesterol efflux in neurons, further contributing to intracellular cholesterol accumulation (18–27). We reported that prion infection affects the major cholesterol efflux pathway, which is mediated by the brain-specific cholesterol-metabolizing enzyme, cholesterol 24-hydroxylase (CYP46A1) (28). CYP46A1 is a member of the cytochrome 450 enzyme family and distributed through different regions of the brain, such as hippocampus, cortex, striatum, thalamus, hypothalamus, and cerebellum. It is primarily expressed in neuronal cells and considered the main enzyme with a primary role in neuronal cholesterol metabolism (29–31)

Efforts to inhibit cholesterol synthesis using compounds like lovastatin and squalestatin have demonstrated reductions in PrP^Sc^ formation in prion-infected cells (18, 19, 32). Similar effects on PrP^Sc^ levels were observed upon treatment with drugs like amphotericin B, which disrupt cholesterol binding in lipid rafts (33–36). The cholesterol transport inhibitor, U18666A, successfully redistributed cholesterol from the plasma membrane to intracellular compartments in N2a cells, reducing PrP^Sc^ levels by enhancing its degradation (24). As such, cholesterol-modulating drugs represent a promising therapeutic avenue for combating prion diseases.

Recently, we have identified that CYP46A1 is significantly reduced in mouse-adapted scrapie prion–infected mouse brains and neuronal cells and in brains of patients with sCJD (28). Efavirenz (EFV), an FDA-approved anti-HIV drug and allosteric activator of CYP46A1 (37–41), was found to significantly reduce PrP^Sc^ levels in prion-infected neuronal cell models without compromising lipid rafts or PrP^C^ levels. Notably, the administration of EFV extended survival and slowed disease progression in scrapie prion–infected mice. The goal of the current study was to verify the beneficial effects of EFV treatment in a relevant model of human prion disease and to explore the impact on brain cholesterol metabolism. We inoculated tg650 mice overexpressing human PrP^C^ (129MM) intracerebrally with MM1 sCJD prions (42, 43) and started low-dose EFV treatment at 30 and 130 days postinoculation (DPI). EFV treatment significantly extended the survival of mice compared with untreated inoculated controls, even when the drug treatment started late at 130 DPI, encompassing more than 70% of the survival time of nontreated sCJD-infected control mice. Furthermore, biochemical and morphological assessments revealed that EFV treatment in both paradigms significantly reduced levels of PrP^Sc^, cholesterol, and lipid droplets while increasing CYP46A1 levels at the early clinical stage, compared with the nontreated sCJD-infected control group. Similar to EFV treatment, CYP46A1 overexpression in mouse-adapted scrapie prion–infected mouse neuroblastoma N2a cells reduced PrP^Sc^, demonstrating a causal correlation between enhanced CYP46A1 activity and reduced PrP^Sc^ levels. Overall, these findings suggest that EFV, even at low doses and administered at a late stage of the incubation period, exerts beneficial effects in an sCJD model, highlighting its potential as a therapeutic agent for human prion diseases.

## Results

### Oral EFV administration significantly prolongs survival of sCJD-infected tg650 mice.

**Figure 1:**
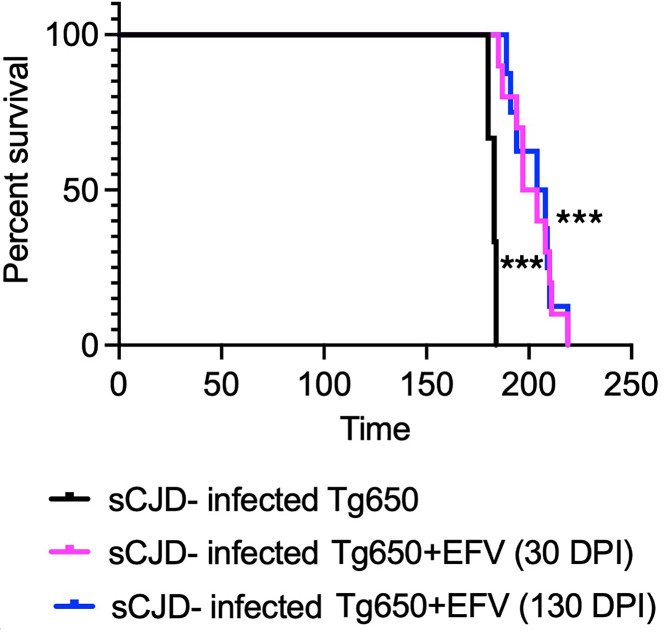
EFV oral administration significantly prolongs survival of sCJD-infected tg650 mice. The Kaplan-Meier plot illustrates the percentage survival of sCJD-infected mice across 3 groups: the untreated group (*n* = 9), the EFV-treated group starting at 30 DPI (sCJD + EFV 30 DPI-DW) (*n* = 10), and the EFV-treated group starting at 130 DPI (sCJD + EFV 130 DPI-DW) (*n* = 10). Log-rank (Mantel-Cox) and Gehan-Breslow-Wilcoxon tests were performed, and statistical significance is ***P < 0.001. DW, drinking water.

Based on our previous findings that EFV treatment significantly prolonged the survival of RML-infected C57BL/6 mice, we extended our study to evaluate the therapeutic effect of oral EFV administration in a mouse model of human prion disease (28). Since many drugs that are effective against scrapie prions and extend survival in animal models fail to work in human prion diseases, it was crucial to validate the efficacy of EFV treatment in the human PrP–overexpressing transgenic tg650 model (42, 43). Tg650 mice were intracerebrally inoculated with sCJD brain homogenate, specifically MM1 sCJD, which we chose because it is the most commonly found sCJD strain. We divided the mice into 3 groups: a nontreated control group (sCJD-tg650), which received no EFV, and 2 treatment groups that received EFV via drinking water at a low dose (1.68 mg/L) used previously in mice to activate Cyp46A1 and that does not have side effects or affect the normal life span of mice of about 700 days (28) (Supplemental Figure 1; supplemental material available online with this article; https://doi.org/10.1172/jci.insight.190296DS1). In the first treatment group, EFV was administered starting at 30 DPI, while in the second group, treatment began at 130 DPI and was continued until the terminal stage of the disease, at which mice exhibited clinical signs of prion disease, including rough coat, tail rigidity, postural instability, ataxia, hunched posture, hind limb clasping, weight loss, gait abnormalities, loss of righting reflex, and unresponsiveness (42). Early clinical signs (rigid tail, rough coat) were observed at 165 DPI in the control group but not in the treatment groups. In both treatment groups, the mice exhibited only early clinical signs (rigid tail, rough coat, hunched posture) at a time point postinfection when the nontreated sCJD-infected group presented with signs of terminal disease (Supplemental Videos 1–3, recorded at 181–182 DPI; Supplemental Tables 1 and 2), indicating slower disease progression. Survival times were recorded, visualized with Kaplan-Meier survival curves, and analyzed using the log-rank (Mantel-Cox) test. Notably, EFV treatment significantly extended the survival of tg650 mice, whether initiated at 30 DPI or 130 DPI, compared with the untreated sCJD-infected group. EFV treatment starting at 30 DPI extended survival by a mean of 17 ± 3.19 days, while treatment initiated at 130 DPI resulted in an even longer extension of 23 ± 4.01 days (Figure 1 and Supplemental Table 3). Notably, we calculated Cohen’s *d* values for effect size of the differences in survival times between control group and 30 DPI, and control group versus 130 DPI treatment groups, which are 2.37 and 3.19, respectively. Values greater than 0.8 are considered very high, indicating a meaningful extension of survival times. These results indicate that EFV may be effective even when administered late in the disease progression.

### EFV reduces PrP^Sc^ accumulation at the early clinical stage in sCJD-infected tg650 mice.

**Figure 2:**
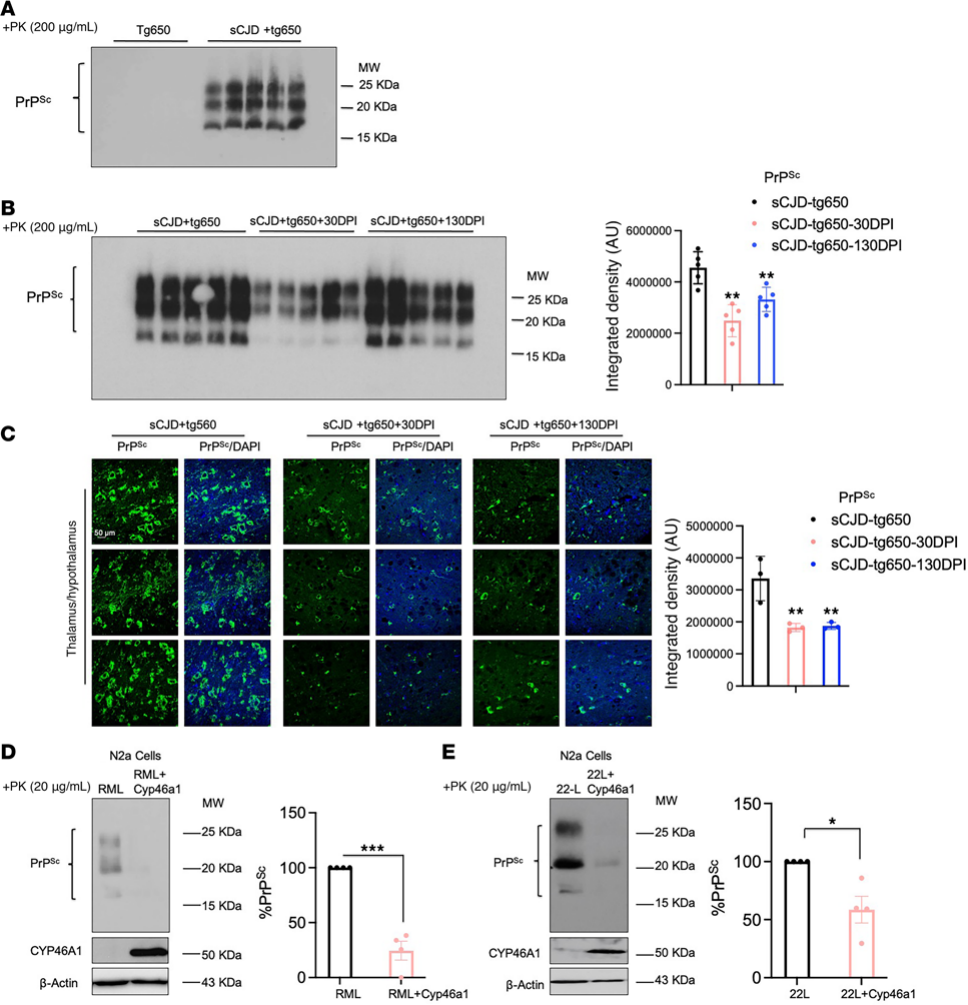
EFV reduces PrP^res^ accumulation at the early clinical stage in sCJD-infected tg650 mice. (**A**) Analysis of uncropped immunoblot of PrP^res^ using the 3F4 antibody in brain homogenates (BH) from 5 noninfected tg650 mice (mock) and sCJD-tg650 mice at the terminal stage. (**B**) Analysis of uncropped immunoblotting and quantification of PrP^res^ using 3F4 antibody in BH from 5 different mice/group at the early clinical stage (176 DPI) for all experimental groups including nontreated and treated (EFV treatment started at 30 DPI and 130 DPI) groups. The histograms are represented as the means ± SEM (*n* = 5 mice/group) of 3 independent experiments. Ordinary 1-way ANOVA was performed, and statistical significance is **P < 0.0092. (**C**) Immunofluorescence and quantification of PrP^res^ using 3F4 antibody in brain tissue from 3 different mice/group at the early clinical stage (176 DPI) for all experimental groups including nontreated and treated (EFV treatment started at 30 DPI and 130 DPI) groups. Fluorescence intensity was quantified; the histograms represent the means ± SEM of *n* = 3 mice per group, obtained from 3 independent experiments. Ordinary 1-way ANOVA was performed, and statistical significance is **P < 0.0062. Original magnification: 63×. Scale bar: 50 μm. (**D** and **E**) Immunoblot analysis of CYP46A1 and PrP^res^ as well as quantification of PrPres in N2a-RML and N2a-RML-*Cyp46a1* overexpression models. The histograms represent the means ± SEM for *n* = 4 per group, obtained from 3 independent experiments. *T* test (2-tailed unpaired *t* test) was performed, and statistical significances are ***P < 0.001 and *P < 0.05, respectively.

At the terminal disease stage, we have observed previously that pathological changes and PrP^Sc^ accumulation did not differ between EFV-treated and nontreated mice (28). Consequently, here we focused our analyses of brain tissue on the early clinical stage of the disease (176 DPI; Supplemental Tables 1 and 2), where effects of therapeutic interventions on PrP^Sc^ levels and other markers may still be detectable. We optimized conditions for detecting PK-resistant PrP^Sc^ (PrP^res^) and determined that a concentration of 200 μg/mL was sufficient to verify the presence of PrP^res^ in sCJD-tg650 mice, as compared with noninfected controls (Figure 2A). Subsequently, we assessed PrP^res^ levels at 176 DPI across all experimental groups. Our data show a significant reduction in PrP^res^ in both EFV treatment paradigms, initiated at 30 DPI and 130 DPI, relative to the untreated sCJD-tg650 group (Figure 2B), indicating that the treatment indeed reduces PrP^res^ propagation. To corroborate these findings and further examine PrP^res^ accumulation in brain regions most affected in sCJD, we performed immunofluorescence analysis. These experiments revealed reduced PrP^res^ reactivity and accumulation in the mediodorsal regions of the thalamus and hypothalamus in treated groups compared with untreated sCJD-tg650 mice (Figure 2C). To assess the effect of EFV at the terminal stage of prion disease, we analyzed samples from all groups at the terminal-stage endpoints. No differences in the PrP^res^ levels were observed between the sCJD-tg650 control and treated groups (Supplemental Figure 2), indicating and validating that terminal-stage PrP^res^ analyses may not provide conclusive insights into the mode of action of potential therapeutic compounds. Similarly, real-time quaking-induced conversion (RT-QuIC) assay was performed on early clinical-stage samples (176 DPI), but no differences in seeding activity were detected (Supplemental Figure 3), suggesting that RT-QuIC might be more informative during the early stages of treatment and preclinical stage of the infected mice.

Next, to explore whether these effects were mediated by the activation of CYP46A1, we conducted in vitro experiments using RML- and 22L-infected N2a cells, with or without overexpression of CYP46A1. Notably, overexpression of CYP46A1 in both RML- and 22L-infected N2a cells significantly reduced PrP^res^ levels compared with nonoverexpressing RML- and 22L-infected N2a cells (Figure 2, D and E). Collectively, these in vivo and in vitro results indicate that EFV exerts its beneficial effects on PrP^res^ reduction primarily through the activation of CYP46A1.

### Higher CYP46A1 and 24S-hydroxycholesterol level in the brain and serum of sCJD-infected tg650 mice as well as in an in vitro model upon EFV treatment.

**Figure 3:**
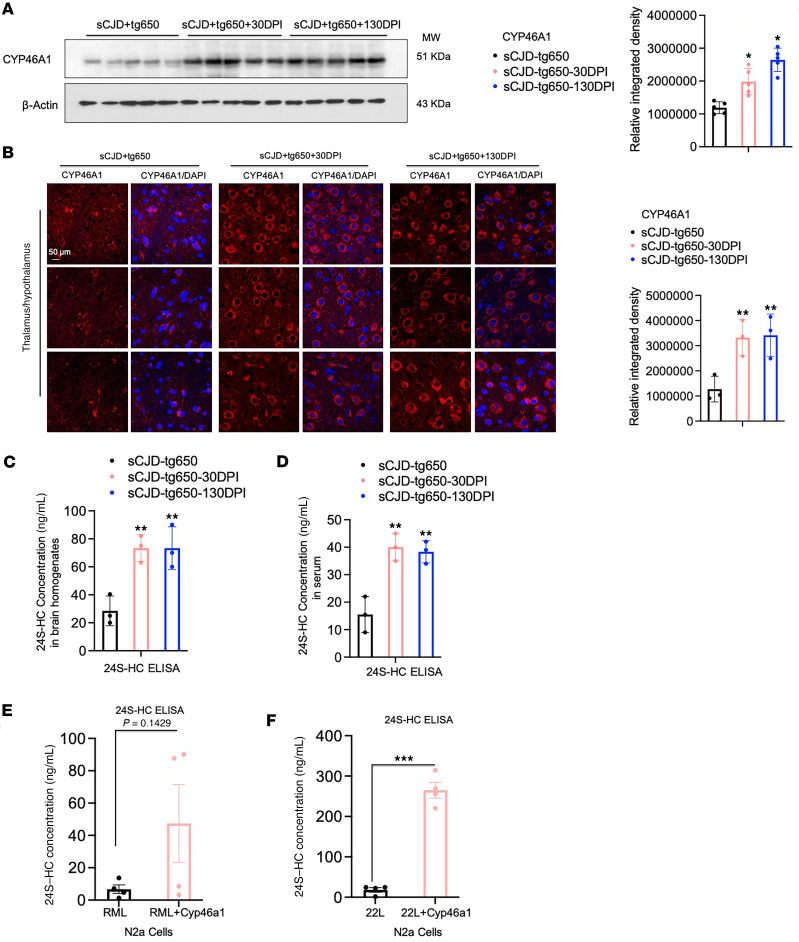
EFV increases CYP46A1 and 24S-HC level in brain and serum of sCJD-infected tg650 mice as well as in an in vitro model. (**A**) Immunoblotting and quantification of CYP46A1 were performed on BH from 5 sCJD-tg650 mice at 176 days DPI, representing the early clinical stage, from control as well as 30 DPI and 130 DPI treatment groups. Histograms show the means ± SEM (*n* = 5 mice/group) based on 3 independent experiments. Ordinary 1-way ANOVA was performed, and statistical significance is *P < 0.0224. (**B**) Immunofluorescence and quantification of CYP46A1 in brain tissue from 3 sCJD-tg650 mice and samples from 30 DPI and 130 DPI treatment groups at 176 DPI (early clinical stage). Fluorescence intensity was quantified, with histograms representing the means ± SEM for *n* = 3 mice/group, gathered from 3 independent experiments. Ordinary 1-way ANOVA was performed, and statistical significance is **P < 0.0161. Magnification: 63×; scale bar: 50 μm. (**C** and **D**) 24S-hydroxycholesterol (24S-HC) quantified by ELISA in BH and serum from sCJD-tg650 control mice and treated groups at the early clinical stage. Histograms represent means ± SEM (*n* = 3 mice/group) from 3 independent experiments. Ordinary 1-way ANOVA was performed, and statistical significances are **P < 0.0057 (**C**) and **P < 0.0022 (**D**). (**E** and **F**) 24S-HC levels quantified by ELISA in the media of N2a-RML and N2a-22L cells, with and without CYP46A1 overexpression. The histograms represent the means ± SEM for *n* = 4 per group, obtained from 3 independent experiments. *T* test (2-tailed unpaired *t* test) was performed, and statistical significances are *P* < 0.1429 (**E**) and ***P < 0.001 (**F**).

Consistent with our previous findings in mouse-adapted scrapie prion–infected mice (28), we observed a significant reduction in CYP46A1 levels at the terminal stage in sCJD-tg650 mice compared with noninfected tg650 controls (Supplemental Figure 4). We next analyzed brain homogenates from untreated and treated groups at the early clinical stage (176 DPI). Notably, CYP46A1 levels were significantly reduced in untreated sCJD-tg650 mice compared with both treatment groups, which received EFV starting at either 30 or 130 DPI (Figure 3A). These results were further supported by immunofluorescence analysis, which also demonstrated a marked reduction in CYP46A1 signal in untreated sCJD-tg650 mice relative to the 2 EFV-treated (at either 30 or 130 DPI) groups at 176 DPI (Figure 3B), possibly related to a less advanced clinical stage in EFV-treated mice. Nevertheless, to determine whether changes in CYP46A1 expression were associated with reactive astrocytes, as previously proposed (44–46), we assessed its colocalization with the astrocytic marker glial fibrillary acidic protein (GFAP). The data indicate that EFV treatment did not increase CYP46A1 expression in reactive astrocytes (Supplemental Figure 5).

Furthermore, to determine if EFV increased the cholesterol metabolite 24S-hydroxycholesterol (24S-HC), which can cross the blood-brain barrier (BBB) and enter peripheral circulation, we performed 24S-HC ELISAs on both brain homogenates and serum samples. EFV treatment in both paradigms significantly increased 24S-HC levels compared with untreated sCJD-tg650 mice (Figure 3, C and D). We also assessed 24S-HC levels in noninfected FVB mice and FVB mice infected with RML and 22L mouse-adapted scrapie prions, finding reduced 24S-HC in the brain homogenates of the infected groups compared with noninfected controls (Supplemental Figure 6). Last, we measured 24S-HC levels in the media of N2a-RML, N2a-22L, and CAD-5-22L cells with and without CYP46A1 overexpression. The ELISA results showed elevated 24S-HC levels in CYP46A1-overexpressing cells infected with RML and 22L prions (Figure 3, E and F, and Supplemental Figure 7), verifying the increase in CYP46A1 enzymatic activity and further supporting its role in cholesterol metabolism under prion infection conditions.

### EFV treatment prevents SREBF2 activation and increases in lipid droplet marker in the brains of sCJD-infected tg650 mice.

**Figure 4:**
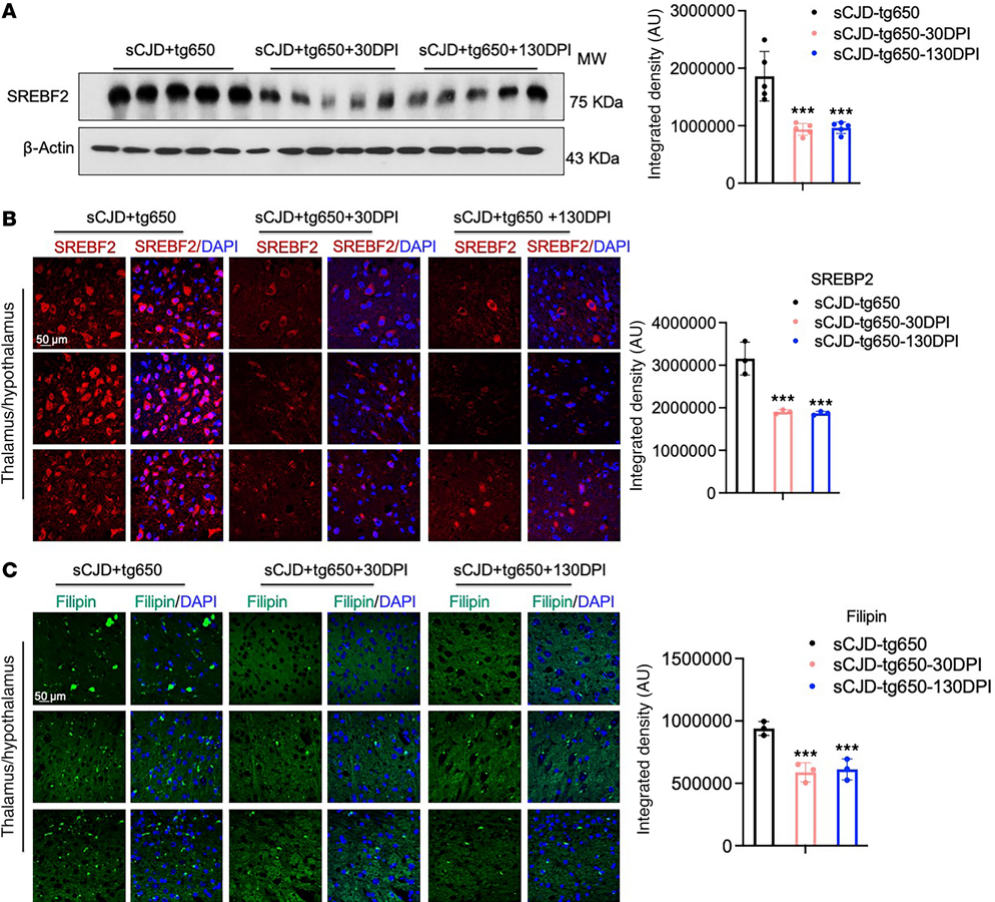
EFV treatment downregulates SREBF2 in the brains of sCJD-infected tg650 mice. (**A**) Immunoblotting and quantification of SREBF2 were conducted on BH from 5 sCJD-tg650 mice at 176 DPI (early clinical stage) as well as in 30 DPI and 130 DPI treatment groups. Histograms show means ± SEM (*n* = 5 mice/group) from 3 independent experiments. Ordinary 1-way ANOVA was performed, and statistical significance is ***P < 0.0002. (**B**) Immunofluorescence staining and quantification of SREBF2 (red) and DAPI (blue) in brain tissue from 3 sCJD-tg650 mice, including samples from 30 DPI and 130 DPI treatment groups at the early clinical stage (176 DPI). Fluorescence intensity was measured, with histograms showing means ± SEM (*n* = 3 mice/group) from 3 independent experiments. Ordinary 1-way ANOVA was performed, and statistical significance is ***P < 0.0007. Magnification: 63×; scale bar: 50 μm. (**C**) Filipin staining and quantification in brain tissue from 3 sCJD-tg650 mice, as well as 30 DPI and 130 DPI treatment group samples at the early clinical stage (176 DPI). Fluorescence intensity was measured and represented as histograms showing means ± SEM (*n* = 3 mice/group) from 3 independent experiments. Ordinary 1-way ANOVA was performed, and statistical significance is ***P < 0.0018. Magnification: 63×; scale bar: 50 μm.

**Figure 5:**
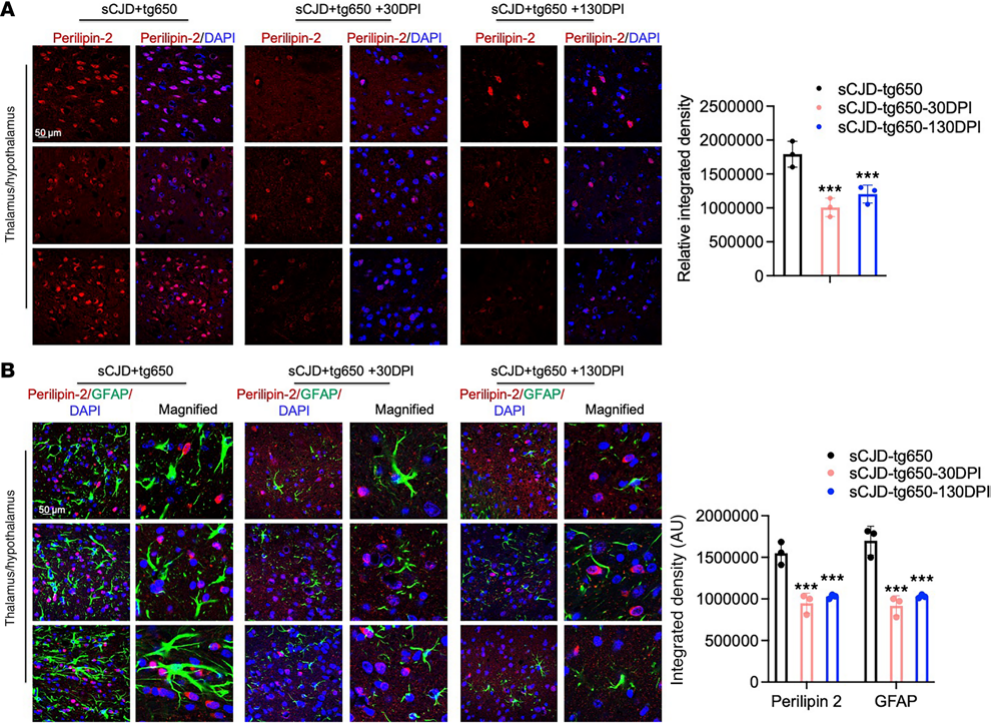
EFV treatment downregulates lipid droplet protein in the brains of sCJD-infected tg650 mice. (**A**) Immunofluorescence staining and quantification of perilipin-2 (red) and DAPI (blue) in brain tissue from 3 sCJD-tg650 mice, including samples from control (nontreated group), 30 DPI and 130 DPI treatment groups at the early clinical stage (176 DPI). Fluorescence intensity was measured, with histograms showing means ± SEM (*n* = 3 mice/group) from 3 independent experiments. Ordinary 1-way ANOVA was performed, and statistical significance is ***P < 0.0020. Magnification: 63×; scale bar: 50 μm. (**B**) Double-immunofluorescence staining for perilipin-2 (red) and GFAP (green) and quantification in brain tissue from 3 sCJD-tg650 mice, as well as 30 DPI and 130 DPI treatment group samples at the early clinical stage (176 DPI). Fluorescence intensity was measured and represented as histograms showing means ± SEM (*n* = 3 mice/group) from 3 independent experiments. Two-way ANOVA was performed, and statistical significances is ***P < 0.0001 for perilipin-2 and GFAP. Magnification: 63×; digital zoom: 2×; scale bar: 50 μm.

Cholesterol is locally synthesized in the brain, and its efflux from the brain is primarily mediated by CYP46A1. Scrapie prions are known to increase the expression of cholesterogenic genes and transcription factor, including SREBF2, and elevate free cholesterol levels in both cells and brain tissue (19–23). Our immunoblotting results verified a significant increase in SREBF2 levels in the brains of sCJD-infected tg650 mice compared with noninfected controls (Supplemental Figure 8A). In contrast, immunoblotting and confocal microscopy results showed that EFV treatment markedly reduced the truncated, activated SREBF2 protein levels in brains compared with untreated sCJD-tg650 mice (Figure 4, A and B). Filipin staining revealed a reduction in cholesterol accumulation in the EFV-treated groups, as indicated by the decreased filipin signal (Figure 4C). Cholesterol esters and other lipids accumulate as lipid droplets, prompting us to assess lipid droplet content of brains by analyzing the lipid droplet–associated protein, perilipin-2, in sCJD-tg650 nontreated and EFV-treated groups. Immunofluorescence analysis revealed that EFV treatment significantly reduced perilipin-2 levels compared with untreated sCJD-tg650 mice (Figure 5A). To determine whether these lipid droplets were associated with astrocytes, we performed double-immunofluorescence staining using perilipin-2 and GFAP. Our results showed that the increased perilipin-2 signal was not associated with reactive astrocytes (Figure 5B). It may be associated with neuronal cells, as our and others’ previous studies have shown that prion infection predominantly leads to cholesterol accumulation in neurons (19, 23, 26, 27). Additionally, we analyzed free cholesterol accumulation using filipin staining in FVB mice. The filipin signal was increased in the brains of RML and 22L scrapie prion–infected mice compared with noninfected (mock) controls (Supplemental Figure 8B). We propose that cholesterol and lipid droplets accumulate in prion-infected mouse brains. EFV has the potential to regulate these, which is likely linked to the activation and elevated expression of CYP46A1, compared with infected, nontreated control mice. This helps maintain cellular cholesterol homeostasis, reduce PrP^Sc^ propagation, and mitigate the detrimental effects of prion infection.

## Discussion

Despite significant efforts, no effective treatments or preventive strategies have been identified to cure prion diseases. Some therapeutic agents, such as pentosan polysulfate and quinacrine, have been investigated in human prion diseases for their ability to inhibit the conversion of PrP^C^ into PrP^Sc^, as they showed promise in prion-infected mouse models as well as reduction of scrapie- and sCJD-PrP^Sc^ in vitro (47–54). However, clinical trials with these drugs in patients with sCJD failed to demonstrate significant improvements in symptoms or survival periods. Doxycycline has been reported to extend survival in PrP^Sc^-inoculated mice, but its efficacy in humans seems limited to early-stage sCJD (53). Recently, Mead and colleagues reported on a distinct investigation where 6 patients with CJD were treated with a monoclonal antibody targeting PrP, administered under a special license (55). Similarly, other studies have highlighted the potential of antisense oligonucleotides and RNA interference as therapeutic strategies for targeting PrP^C^ expression to treat prion diseases (56–59). The challenges of developing therapeutics for sCJD are multifaceted. First, antiprion drugs demonstrate the highest efficacy in animal models when administered concurrently with or early after prion infection (49–60). Unfortunately, human trials begin after disease onset, as early diagnostic tools are still lacking. Second, drug effects vary depending on the prion strain, and most importantly, the majority of the drugs do not effectively cross the BBB (60–63). Prions exhibit strain diversity, and the mechanisms behind this diversity and strain specificity of antiprion compounds remain poorly understood (63). For example, the RML strain, a mouse-adapted scrapie prion, is frequently used in therapeutic testing but differs significantly from human sCJD prions in its biochemical and pathological properties. Even if a compound extends the survival of scrapie prion–inoculated mice, it may not necessarily be effective against human prions (63–65). Thus, adopting a disease-modifying and unified approach could be a more promising strategy to identify therapeutic tools and test drugs in human prion–inoculated transgenic models overexpressing human PrP. EFV is a non-nucleoside reverse transcriptase inhibitor that has been widely used for decades in the chronic treatment of HIV infection. This drug efficiently crosses the BBB and has been approved by the FDA as an anti-HIV therapy since 1998 (37, 38). Our studies demonstrate therapeutic potential in a model of scrapie prions (28), and more importantly here, in a model of human sCJD. EFV’s ability to prolong survival and modulate prion-related pathologies in sCJD-infected tg650 mice marks an important advancement in prion disease research, particularly because many drugs that are effective in scrapie prion models fail in human prion diseases. Here, we provide compelling evidence that EFV’s beneficial effects in tg650 mice are mediated through mechanisms involving cholesterol metabolism and homeostasis, specifically through the activation of the brain cholesterol-metabolizing enzyme CYP46A1.

PrP^Sc^ accumulation is a hallmark of prion diseases, and its presence is associated with neurodegeneration and the spread of prion pathology (1–8). A growing body of evidence suggests that the accumulation of PrP^Sc^/PrP^res^, the misfolded form of the prion protein, in prion-infected cells and animal models is closely linked to the upregulation of cellular cholesterol (17–26). There is substantial evidence indicating that prion infection elevates cholesterol levels through increased cholesterol synthesis and reduced efflux (23, 24, 26–28). Aberrantly increased cholesterol may enhance prion conversion efficiency and disrupt vesicle trafficking, particularly within the endolysosomal pathway (25, 26). Impaired cholesterol metabolism is a recognized feature in various neurodegenerative diseases (Alzheimer’s disease [AD], Parkinson’s disease [PD], Huntington’s disease, and Niemann-Pick disease type C), where it promotes the accumulation of misfolded proteins forming toxic aggregates. This dysregulation may represent a common pathological mechanism across neurodegenerative diseases linked to protein misfolding (66–68). In prion research several compounds and drugs were used to inhibit cholesterol synthesis, cholesterol accumulation, or its regulation as well as inhibition or degradation of PrP^Sc^ (69–73). However, these therapeutics were useful to extend the survival of mice, but none of these studies showed significant reductions in PrP^Sc^ accumulation or cholesterol regulation, suggesting that the survival benefit was not due to cholesterol regulation. Similarly, other cholesterol-depleting agents, such as methyl-beta cyclodextrin, reduced PrP^Sc^ in infected cells (74, 75), but these treatments lower cellular cholesterol below normal and basal levels, which can affect physiological function, disrupting membrane integrity and causing cellular dysfunction. Brain cholesterol plays a crucial role in various cellular processes, including the regulation of neurotransmitters, cellular trafficking, synapse formation, and synaptic and cognitive functions (31, 76–78). Furthermore, cholesterol is essential for producing biologically active compounds, such as neurosteroids and oxysterols, which are involved in key physiological and cellular pathways (31, 79). Thus, cholesterol depletion may not be an ideal therapeutic approach for prion diseases. Instead, targeting cellular pathways that modulate cholesterol without affecting its basal levels is crucial. Notably, around 45%–50% of brain cholesterol turnover is regulated by the brain-specific enzyme CYP46A1 (80–85), offering a disease-modifying approach by regulating cholesterol homeostasis that emerged as a potential therapeutic strategy for neurodegenerative diseases, including prion diseases. Our work highlights the importance of CYP46A1, a cholesterol-metabolizing enzyme, and its activation by EFV, an FDA-approved antiretroviral drug (37, 38), in reducing prion pathology. Earlier we showed that CYP46A1 levels are significantly reduced in scrapie prion–infected mice and neuronal cells and brain of patients with sCJD. EFV demonstrated its ability to lower PrP^Sc^ levels in various prion-infected, immortalized neuronal and primary cerebellar granular neuron cell models and extended survival in scrapie prion–infected mice without affecting lipid raft membrane integrity or PrP^C^ levels (28). A key finding of our current study is the upregulation of 24S-HC by EFV in in vitro models and sCJD-tg650 mice, which plays a critical role in maintaining cholesterol homeostasis in the brain, and validation that EFV treatment is effective against human prions. Prion infections are known to disrupt cholesterol metabolism, leading to lipid dysregulation and exacerbation of neurodegeneration (Supplemental Figure 4) (17–24, 28). Upon EFV treatment, starting at both 30 DPI and 130 DPI, unlike in the control group, we did not observe a significant reduction of CYP46A1 levels in the brain, as verified by immunoblotting and immunofluorescence (Figure 3, A and B), similar to other studies reporting that EFV treatment increases CYP46A1 activity and even expression (86, 87). While in our study, CYP46A1 expression in brains of EFV-treated mice did not localize to activated astrocytes, the overall level of activated astrocytes was lower, raising the question whether EFV treatment influences astrocyte activation; however, effects of EFV on reactive astrocytes remain controversial. For instance, chronic EFV treatment in 5xFAD mice increased GFAP levels (86), whereas in the M83 transgenic mouse model of PD injected with preformed fibrils, EFV treatment elevated CYP46A1 levels while reducing reactive astrocytes and microglia (87). Given these diverse and sometimes conflicting findings as well as emerging data from our prion disease models, further investigation is needed to elucidate the mechanisms by which chronic EFV treatment modulates CYP46A1 levels and how this influences glial cell biology.

Notably, EFV treatment led to elevated levels of 24S-HC in both brain and serum (Figure 3, C and D). This metabolite, which crosses the BBB and enters the peripheral circulation, underscores the critical role of CYP46A1 in cholesterol turnover. This effect is further reflected in reduced cholesterol levels through the regulation of SREBF2 and lipid accumulation, as evidenced by the decreased expression of the lipid droplet marker perilipin-2. Elevated 24S-HC levels were noted in brain and serum samples from EFV-treated sCJD-tg650 mice, further supporting the hypothesis that CYP46A1 activation is a central mechanism by which EFV exerts its therapeutic effects in prion-infected brains.

Most importantly, EFV treatment led to a significant reduction in PrP^Sc^ levels at the early clinical stage in sCJD-infected tg650 mice (Figure 3, B and C). This reduction in PrP^res^ may be linked to the activation of CYP46A1, which we demonstrated in vitro through CYP46A1 overexpression experiments in RML- and 22L-infected N2a cells. The reduction of PrP^res^ at the early clinical stage suggests that EFV may slow disease progression, delay the onset of clinical signs, and extend the survival in sCJD-infected tg650 mice. In both treatment paradigms (initiated at 30 days DPI and 130 DPI), EFV extended survival by 17 and 23 days, respectively, compared with untreated mice (Figure 1). This indicates that EFV remains effective even when administered during later stages of disease progression — a crucial consideration for human prion diseases, particularly sCJD, where patients are diagnosed at an early clinical stage. EFV was administered at minimal doses (0.09 mg/kg/d orally), about 300–400 times lower than the standard dosage for patients with HIV, without causing any observable side effects. At these low doses, EFV effectively activates CYP46A1 in mice and has demonstrated significant efficacy across various animal models of neurodegenerative diseases (28, 39–41, 68). EFV has recently demonstrated success in clinical trials for patients with AD. In these trials, low doses (50 and 200 mg daily for 20 weeks) of EFV were used to activate CYP46A1 in individuals with early-stage AD (88), suggesting its potential as a therapeutic approach for neurodegenerative diseases. Taken together, these findings indicate that EFV may have some potential against human prion diseases, including sCJD, warranting further exploration of this treatment.

## Methods

### Sex as a biological variable.

Our study exclusively examined female mice, as there are no reported sex differences in prion disease pathogenesis or susceptibility. Due to the extended duration of our experiments, male mice develop fighting behavior; therefore, females are preferred.

### Animals.

Female transgenic tg650 mice overexpressing human PrP^C^ (129MM) (42) from our in-house colonies were used in this bioassay. The mice were maintained on a 12-hour light/12-hour dark cycle at a controlled temperature of 23°C, with 60% ± 10% humidity. Food and water were provided ad libitum.

### Chemicals and antibodies.

EFV was obtained from Toronto Research Chemicals Inc. The PK and protease inhibitor (Pefabloc) were purchased from Roche. All other reagents and chemicals were purchased from Sigma-Aldrich. The mouse monoclonal antibody 4H11 used in this study to detect PrP has been previously described (89). Other antibodies used included rabbit CYP46A1 polyclonal antibody (12486-1-AP), rabbit SREBP2 polyclonal antibody (14508-1-AP), and rabbit ADRP/Perilipin-2 polyclonal antibody (15294-1-AP) from Proteintech, along with the mouse 3F4 monoclonal antibody (66369-1-Ig), which recognizes amino acid residues 109–112 of human PrP, from BioLegend. Mouse monoclonal GFAP antibody (2E1) (sc-33673) was purchased from Santa Cruz Biotechnology, and mouse monoclonal β-actin antibody (A5441) was purchased from Sigma-Aldrich. The secondary goat anti-mouse HRP-conjugated antibody was sourced from Jackson ImmunoResearch.

### Mouse bioassay.

After acclimatization, tg650 mice were randomly assigned into 3 groups of *n* = 15 in each group: (a) sCJD (sCJD+tg650), no treatment; (b) treatment paradigm 1, sCJD-inoculated tg650 + EFV (30 DPI-DW), with EFV supplied in DW starting at 30 DPI; and (c) treatment paradigm 2, sCJD-inoculated tg650 + EFV (130 DPI-DW), with EFV supplied in DW starting at 130 DPI (Supplemental Tables 1 and 2). At 6–8 weeks of age, all mice were intracerebrally (i.c.) inoculated under anesthesia into the right parietal lobe with 20 μL of 1% sCJD (MM1) brain homogenates using a 25-gauge disposable hypodermic needle. The mice were carefully monitored daily for 1 week following i.c. injection. EFV (1.68 mg/L) treatment in drinking water was started either at 30 (treatment paradigm 1) or at 130 DPI (treatment paradigm 2). EFV was dissolved in 0.01% DMSO and added to the DW. Every 3 days the EFV solution in DW was replaced with fresh EFV solution, continuing until the experimental endpoint. The mice were monitored weekly, then daily when clinical signs progressed. Masked assessments were conducted by animal care staff and, for confirmation, by research personnel. At 176 DPI, mice in the nontreated group exhibited moderate to severe clinical signs of prion disease, including rough coat, rigid tail, imbalance, ataxia, hunched posture, and weight loss. In contrast, mice in both treatment groups displayed only mild signs at the same time point (Supplemental Table 1). We euthanized 5 mice from each group for biochemical and immunohistochemical analyses (Supplemental Figure 1). The remaining mice were euthanized under anesthesia at terminal disease stage (Supplemental Figure 2), and survival times were recorded (Figure 1). One mouse from the nontreated group was euthanized for intercurrent diseases and was excluded from statistical analyses.

### Preparation of brain homogenates.

Brain homogenates were prepared in PBS (20% w/v) using a FastPrep 24 (MP Biomedicals) for 3 cycles each with 1-minute duration. The homogenates were aliquoted and stored at –80°C until further use. For immunoblotting, 20% brain homogenate was mixed with equal volume of cold lysis buffer (10 mM Tris-HCl at pH 7.5, 100 mM NaCl, 10 mM EDTA, 0.5% Triton X-100, 0.5% sodium deoxycholate). Resulting 10% brain homogenates were incubated with solvent for no PK (–PK) or 200 μg/mL of PK (+PK) and digested at 37°C for 1 hour. PK digestion was stopped by addition of proteinase inhibitors (Pefabloc). This was followed by addition of 3× sample loading buffer and boiling at 95°C for 5–7 minutes. The samples were processed for immunoblotting as described below.

### Immunoblotting.

Immunoblot analysis was performed as previously described (28). Protein samples were resuspended in TNE buffer (50 mM Tris-HCl, pH 7.5; 150 mM NaCl; 5 mM EDTA) and separated on 12.5% SDS-PAGE. Electroblotting was done using Amersham Hybond P 0.45 PVDF membranes. Membranes were incubated with primary and secondary antibodies as indicated in the following section and analyzed using Luminata Western Chemiluminescent HRP Substrates (MilliporeSigma). The densitometric analysis of immunoblots was performed using ImageJ (NIH).

### Immunofluorescence staining and confocal microscopy.

Paraffin-embedded mouse brain hemisphere tissue samples (5 μm–thick sections) on gelatin-coated slides were deparaffinized 2 times with fresh absolute xylene (5 minutes for each wash) and rehydrated with graded ethyl alcohol (100%, 90%, 80%, and 70%). After rehydration these slides were washed twice with water and TBST (10 mM Tris-HCl at pH 7.4, 150 mM NaCl, 0.05% Tween 20) for 10 minutes and incubated for 1 hour with 2% goat serum as a blocking solution and 0.3% Triton X-100 in PBS. Then slides were then incubated with primary antibodies (CYP46A1 Polyclonal antibody [12486-1-AP], rabbit SREBP2 Polyclonal antibody [14508-1-AP], rabbit ADRP/Perilipin-2 Polyclonal antibody [15294-1-AP] from Proteintech; Mouse monoclonal GFAP antibody [2E1] sc-33673) from Santa Cruz Biotechnology) diluted 1:100 in blocking solution overnight at 4°C. After primary antibody incubation, the sections were washed twice for 5 minutes each and incubated for 1 hour with Alexa Fluor 488 goat anti-rabbit/mouse or Alexa Fluor 555 goat anti-rabbit/mouse secondary antibodies (1:400) (Jackson ImmunoResearch). Coverslips were mounted using DAPI-mounting medium (Thermo Fisher Scientific).

Filipin staining was performed as described previously (26, 90). Five images per section (tissue) were captured from each respective group at same conditions for all images using a confocal laser scanning microscope (ZEISS LSM 700 confocal microscope). Confocal images were converted to tagged image file format (TIF). The quantification of the immunofluorescence intensity in the same region of the brain areas (thalamus/hypothalamus mediodorsal region) in the TIF images for all groups was performed using ImageJ software. The background of TIF images was optimized according to the threshold intensity, and the immunofluorescence intensity was analyzed at specified threshold intensity for all groups at same conditions and was expressed as the relative integrated density between the groups.

### Cell culture, transfection, and cell lysis.

The murine neuroblastoma cell line N2a was obtained from ATCC (CCL-131). CAD5 and CAD5-22L neuronal cells were a gift from Sukhvir Mahal, Scripps Research Institute, Jupiter, Florida, USA. The N2a cells persistently infected with RML or 22L scrapie prions were cultured in OptiMEM+GlutaMax media (Gibco) containing 10% fetal bovine serum and 1% penicillin-streptomycin at 37°C with 5% CO_2_. Transfections were performed in 6 cm tissue culture plates 24 hours after seeding 1 × 10^6^ cells using Lipofectamine LTX with Plus Reagent kit (Thermo Fisher Scientific) according to the protocol. At 72 hours posttransfection, cells were rinsed with 1× PBS before lysing the cells with 1 mL of cold lysis buffer (10 mM Tris-HCl pH 7.5, 100 mM NaCl, 10 mM EDTA, 0.5% Triton X-100, 0.5% sodium deoxycholate) for 10 minutes. Lysates were centrifuged at 21,000*g* for 1 minute and divided for PK digestion (20 μg/mL of PK; for 30 minutes at 37°C) or precipitation without PK digestion. PK digestion was stopped by the addition of Pefabloc protease inhibitors, followed by methanol precipitation. Samples were further processed as previously described (26, 28).

### 24S-HC ELISA.

To measure the 24S-HC levels in brain, serum, and in vitro media, we applied the 24(S)-hydroxycholesterol ELISA kit (Abcam, ab204530) in accordance with the manufacturer’s protocol.

### RT-QuIC assay.

RT-QuIC was conducted as previously described (91) using recombinant mouse PrP (aa 23–231) as substrate. The reaction mix included 10 μg of recombinant PrP, 10 μM Thioflavin-T, 170 mM NaCl, 1× PBS (containing 130 mM NaCl), 1 mM EDTA, and water. Each well of a 96-well plate (MilliporeSigma, no. 3603) received 98 μL of this mix plus 2 μL of the seed (20% w/v brain homogenate with 0.1% SDS), diluted from 1 × 10^–4^ to 1 × 10^–9^ in quadruplicate. Plates were sealed, incubated at 42°C, and shaken at 700 rpm in a BMG Labtech FLUOstar plate reader for 50 hours. Thioflavin-T fluorescence was measured every 15 minutes, and RFU values were averaged across replicates and graphed using GraphPad software. A sample was deemed positive if 2 of the 4 replicates exceeded the threshold (mean RFU of the negative control plus 5 standard deviations). An age-matched, noninfected tg650 brain served as the negative control.

### Statistics.

Statistical analyses (Supplemental Table 3) and histograms were produced using GraphPad Prism 8 software. For statistical analysis of immunoblot signals, 2-tailed independent *t* test for 2 groups or 1-way ANOVA followed by Tukey’s post hoc test or Dunnett’s multiple-comparison test, as applicable, were used. Values are expressed as mean ± SEM. The graphical representation of survival times of animals was done using a Kaplan-Meier plot. The log-rank test was used for statistical analysis of differences between groups in the survival plot with median and pairwise comparisons between control and treated group. Significance was considered at *P* ≤ 0.05.

### Study approval.

All animal experiments described in this study were approved by the University of Calgary Health Sciences Animal Care Committee (protocol: AC18-0158), in accordance with guidelines issued by the Canadian Council on Animal Care and the Animal Research: Reporting of In Vivo Experiments guidelines.

### Data availability.

Values for all data points in graphs are reported in the Supporting Data Values file.

## Author contributions

TA and SG designed the study, wrote the manuscript, and organized the data. TA conducted the animal bioassay, in vivo immunoblotting, immunofluorescence, and data analysis. SH contributed to mouse inoculation and tissue sectioning, JC conducted in vitro experiments, and HAH contributed to the RT-QuIC assay. HS collaborated on studies involving FVB mice infected with scrapie prions. All authors reviewed, edited, and approved the final version of the manuscript for publication. SG is the corresponding author and assumes full responsibility for the manuscript.

## Supplementary Material

Supplemental data

Unedited blot and gel images

Supplemental video 1

Supplemental video 2

Supplemental video 3

Supporting data values

## Version 1 06/20/2025

In-Press Preview

## Version 2 07/22/2025

Electronic publication
