# Supplementary material for: Treatment with efavirenz extends survival in a Creutzfeldt-Jakob disease model by regulating brain cholesterol metabolism
Source: JCI Insight. 2025 Jun 19;10(14):e190296. doi: 10.1172/jci.insight.190296 (PMC12288963; doi:10.1172/jci.insight.190296)
Supplement: Supplemental data [file jciinsight-10-190296-s051.pdf]

Journal of Clinical Investigation Insights 190296-INS-RG-RV-3

Treatment with efavirenz extends survival in Creutzfeldt-Jakob disease model by regulating brain cholesterol metabolism

Tahir Ali[1-4], Jessica Cashion[1-3], Samia Hannaoui[1-3], Hanaa Ahmed-Hassan[1-3], Hermann M Schatzl[1-3], Sabine Gilch*[1-3]

[1] Calgary Prion Research Unit, Faculty of Veterinary Medicine, University of Calgary, Calgary, Alberta, Canada.

[2] Hotchkiss Brain Institute, Cumming School of Medicine, University of Calgary, Calgary, Alberta, Canada.

[3] Snyder Institute for Chronic Diseases, University of Calgary, Calgary, Canada.

[4] Current affiliation: University of Ottawa Brain and Mind Research Institute, Department of Cellular and Molecular Medicine, University of Ottawa, Ottawa, ON, Canada

Running title: EFV, an FDA-approved medication to treat human prion diseases

*Corresponding author

Sabine Gilch, PhD

Professor

UCalgary Research Excellence Chair, Faculty of Veterinary Medicine

University of Calgary, 3330 Hospital Drive NW Calgary, AB T2N 4Z6, Canada

Office: HRIC1AC66; Phone: (403) 210-7578; E-mail: sgilch@ucalgary.ca

Supplementary tables

Table S1: Details of mice at early clinical stage

**Mouse ID**	**sCJD Inoculation**	**Groups**	**Treatment**	**DPI**	**Early clinical stage**
2HsCJD-1-4065	-	sCJD	No treatment	176	Hunched posture, hindlimb clasping, rigid tail, rough coat, and weight loss
2HsCJD-1-4070	-	-	-	-	-
2HsCJD-1-4072	-	-	-	-	-
2HsCJD-1-4078	-	-	-	-	-
2HsCJD-1-4078	-	-	-	-	-
**Mouse ID**	**sCJD**	**sCJD+30 DPI**	**Oral treatment, EFV in drinking water**	**DPI**	**Early clinical stage**
2HsCJD-1-4110	-	-	-	176	Rigid tail, rough coat, mild hunched posture
2HsCJD-1-4111	-	-	-	-	-
2HsCJD-1-4112	-	-	-	-	-
2HsCJD-1-4120	-	-	-	-	-
2HsCJD-1-4124	-	-	-	-	-
**Mouse ID**	**sCJD**	**sCJD+130 DPI**	**Oral treatment, EFV in**	**DPI**	**Early clinical stage**

			**drinking water**		
2HsCJD-1-4128	-	-	-	176	Rigid tail, rough coat, mild hunched posture, and less weight loss
2HsCJD-1-4130	-	-	-	-	-
2HsCJD-1-4134	-	-	-	-	-
2HsCJD-1-4135	-	-	-	-	-
2HsCJD-1-4130	-	-	-	-	-

Table S2: Details of mice at terminal stage

Mouse ID	sCJD Inoculation	Groups	Treatment	DPI	Terminal stage of prion disease
2HsCJD-1-4066	-	sCJD	No treatment	181	Rough coat, rigid tail, imbalance, ataxia, hunched posture, hindlimb clasping, irresponsiveness, gait abnormalities, loss of righting reflex and weight loss
2HsCJD-1-4068	-	-	-	185	-
2HsCJD-1-4069	-	-	-	181	-
2HsCJD-1-4071	-	-	-	178	-
2HsCJD-1-4073	-	-	-	178	-
2HsCJD-1-4074	-	-	-	180	-
2HsCJD-1-4075	-	-	-	183	-
2HsCJD-1-4076	-	-	-	184	-
2HsCJD-1-4069	-	-	-	185	-
	-				

Mouse ID	sCJD	sCJD+ 30 DPI	Oral treatment, EFV in drinking water	DPI	Terminal stage of prion disease
2HsCJD-1-4112	-	-	-	197	Rough coat, rigid tail, imbalance, ataxia, hunched posture, hindlimb clasping, irresponsiveness, gait abnormalities, loss of righting reflex and weight loss

2HsCJD-1-4113	-	-	-	204	-
24sCJD-1-4114	-	-	-	187	-
2HsCJD-1-4115	-	-	-	197	-
2HsCJD-1-4116	-	-	-	211	-
2HsCJD-1-4117	-	-	-	194	-
2HsCJD-1-4118	-	-	-	208	-
2HsCJD-1-4119	-	-	-	166	Humane end point
2HsCJD-1-4120	-	-	-	210	-
2HsCJD-1-4121	-	-	-	185	-
2HsCJD-1-4123	-	-	-	219	-

**Mouse ID**	**sCJD**	**sCJD+ 130 DPI**	**Oral treatment, EFV in drinking water**	**DPI**	**Terminal stage of prion disease**
2HsCJD-1-4124	-	-	-	206	Rough coat, rigid tail, imbalance, ataxia, hunched posture, hindlimb clasping, irresponsiveness, gait abnormalities, loss of righting reflex and weight loss
2HsCJD-1-4125	-	-	-	210	-

2HsCJD-1-4126	-	-	-	191	-
24sCJD-1-4128	-	-	-	204	-
2HsCJD-1-4130	-	-	-	219	-
2HsCJD-1-4131	-	-	-	194	-
2HsCJD-1-4132	-	-	-	206	-
2HsCJD-1-4135	-	-	-	189	-
2HsCJD-1-4136	-	-	-	208	-
2HsCJD-1-4138	-	-	-	209	-

Table S3: Details of statistical analyses

Figures	Parameters analyzed	Groups	Statistical tests	p-Values	Post hoc analysis
Fig. 1	Animal survival	Non-treated VS treated	Log-rank (Montel-Cox) test	<0.0001	NA
		-	Gehan-Breslow-Wilcoxon test	<0.0001	-
Fig. 2B	Immunoblotting of PrP^{res} (3F4) at pre-clinical stage (176DPI)	-	Ordinary One-way ANOVA	<0.0092	-
Fig. 2C	IF of PrP^{res} (3F4)	-	-	<0.0062	-
Fig. 2D	Immunoblotting of PrP^{res} (4H11) in vitro	-	T-test (Unpaired t test)	<0.001	-
Fig. 2E	Immunoblotting of PrP^{res} (4H11) in vitro	-	T-test (Unpaired t test)	<0.05	-
Fig. 3A	Immunoblotting of CYP46A1	-	Ordinary One-way ANOVA	<0.0224	-
Fig. 3B	IF of CYP46A1	-	-	<0.0161	-
Fig. 3C	Brain 24SHC-ELISA	-	-	<0.0057	-
Fig. 3D	Serum 24SHC-ELISA	-	-	<0.0022	-
Fig. 3E	Media 24SHC-ELISA	-	T-test (Unpaired t test)	<0.1429	-
Fig. 3F	Media 24SHC-ELISA	-	T-test (Unpaired t test)	<0.001	-
Fig. 4A	Immunoblotting of SREBF2	-	Ordinary One-way ANOVA	<0.0002	-
Fig. 4B	IF of SREBF2	-	-	<0.0007	-
Fig. 4C	Filipin staining	-	-	<0.0018	-
Fig. 5A	IF of Perilipin	-	-	<0.0020	
Fig. 5B	IF of Perilipin	-	Two-way ANOVA	<0.0001	-
Fig. 5B	IF of GFAP	-	-	<0.0001	-
Fig. 4S	Immunoblotting of CYP46A1	-	T-test (Unpaired t test)	<0.0273	-
Fig. 6S	Brain 24SHC-ELISA	-	Ordinary One-way ANOVA	<0.0041	-
Fig. 7S	Media 24SHC-ELISA	-	T-test (Unpaired t test)	<0.05	-

Supplementary figures and legends

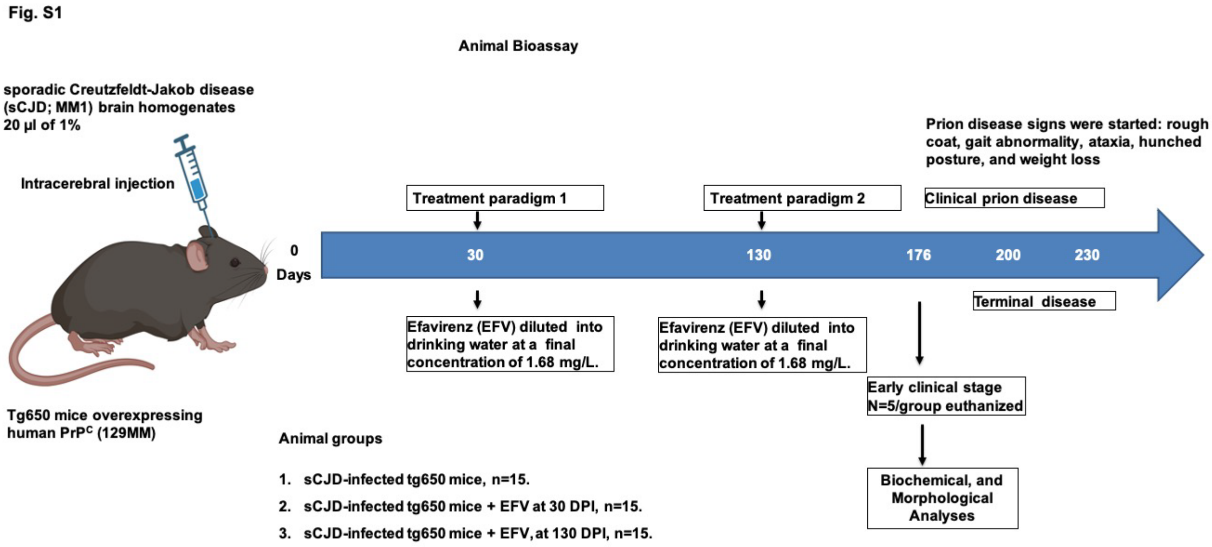

Fig S1. Schematic diagram illustrating the animal bioassay design and Efavirenz (EFV) treatment paradigms. The mouse and syringe images were created using BioRender.

Fig. S2

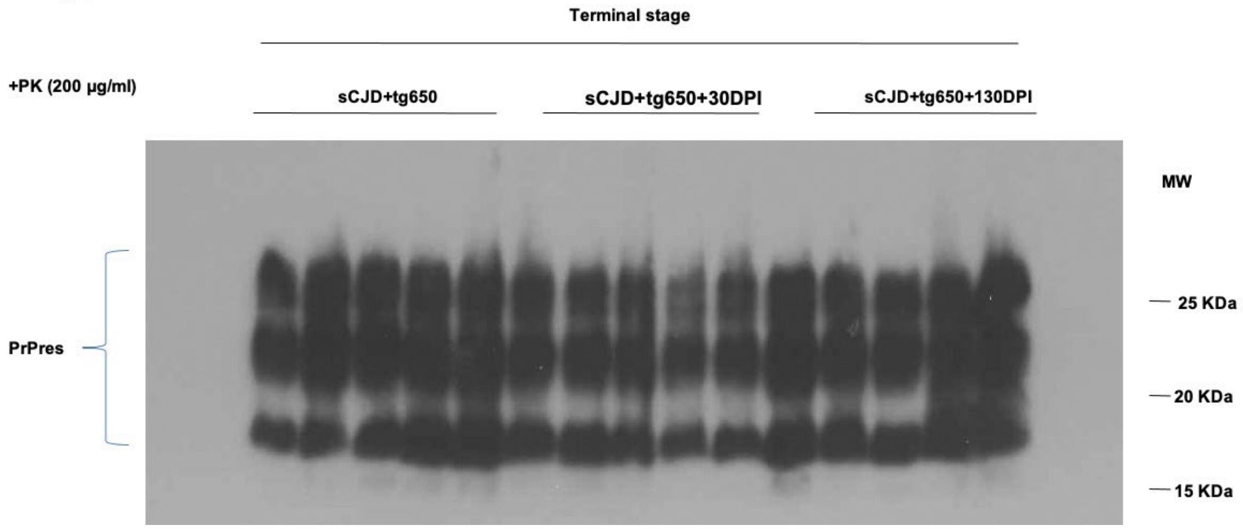

Fig. S2. PrP^{res} levels at the terminal stage of prion disease. Immunoblot analysis of uncropped PrPres using the 3F4 antibody in brain homogenates from non-treated sCJD-tg650 mice, and sCJD-tg650 mice EFV-treated at 30 DPI and 130 DPI.

Fig. S3

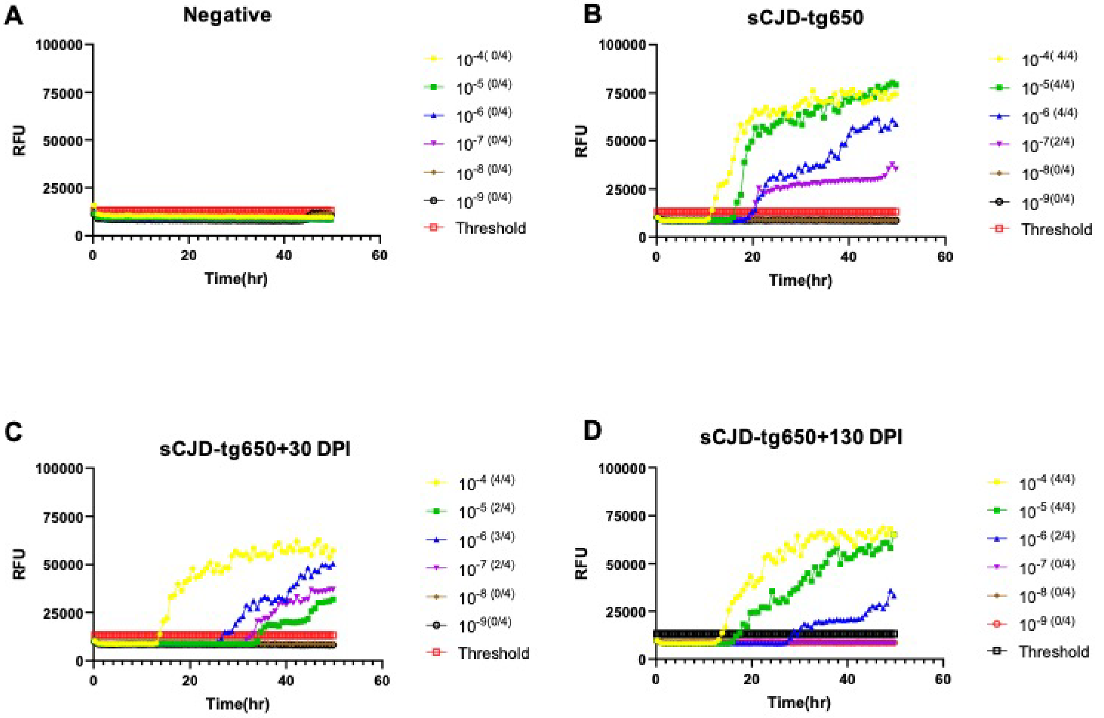

Fig. S3. Representative RT-QuIC graphs illustrating seeding activity in the brain homogenates at the early clinical stage (176 DPI) for all experimental groups including non-treated and treated (EFV treatment started at 30 DPI and 130 DPI) groups. The negative control consisted of brain tissue from age-matched, non-infected tg650 mice. Samples were considered positive if 2 out of 4 wells crossed the threshold, defined as the average RFU of the negative control group plus five times its standard deviation. The y-axis indicates RFU, while the x-axis represents time in hours (hr).

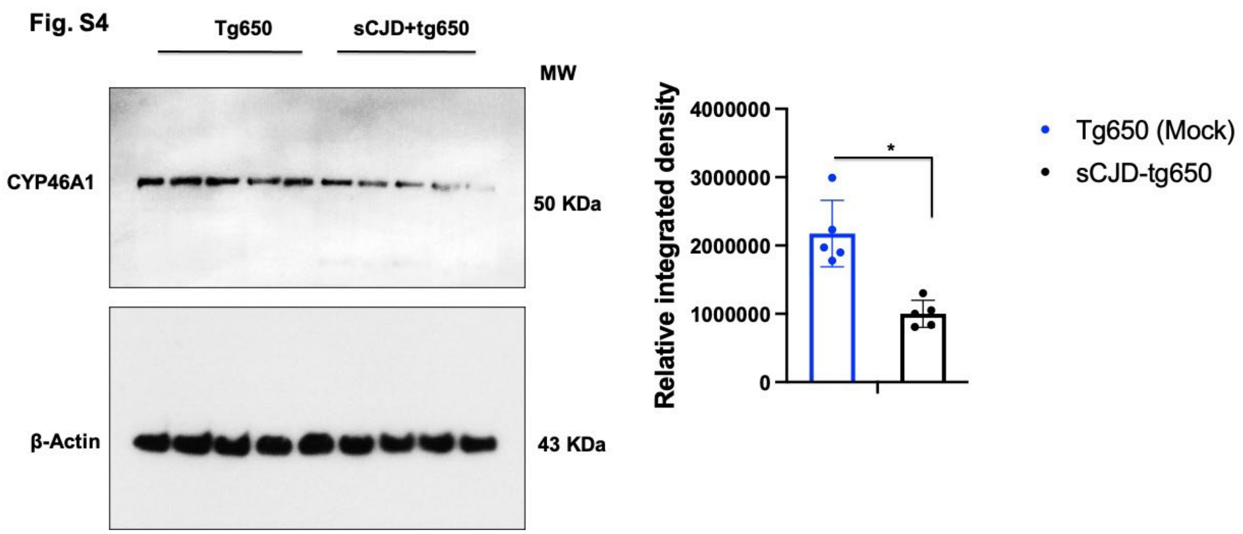

Fig. S4. Analysis of uncropped immunoblot and quantification of CYP46A1 in brain homogenates from five different non-infected (mock) tg650 mice and sCJD-tg650 mice at the terminal disease stage. The histograms are represented as the means ± SEM (n = 5 mice/group) of three independent experiments. T-test (unpaired t test) was performed, and statistical significance is $p<0.0273$.

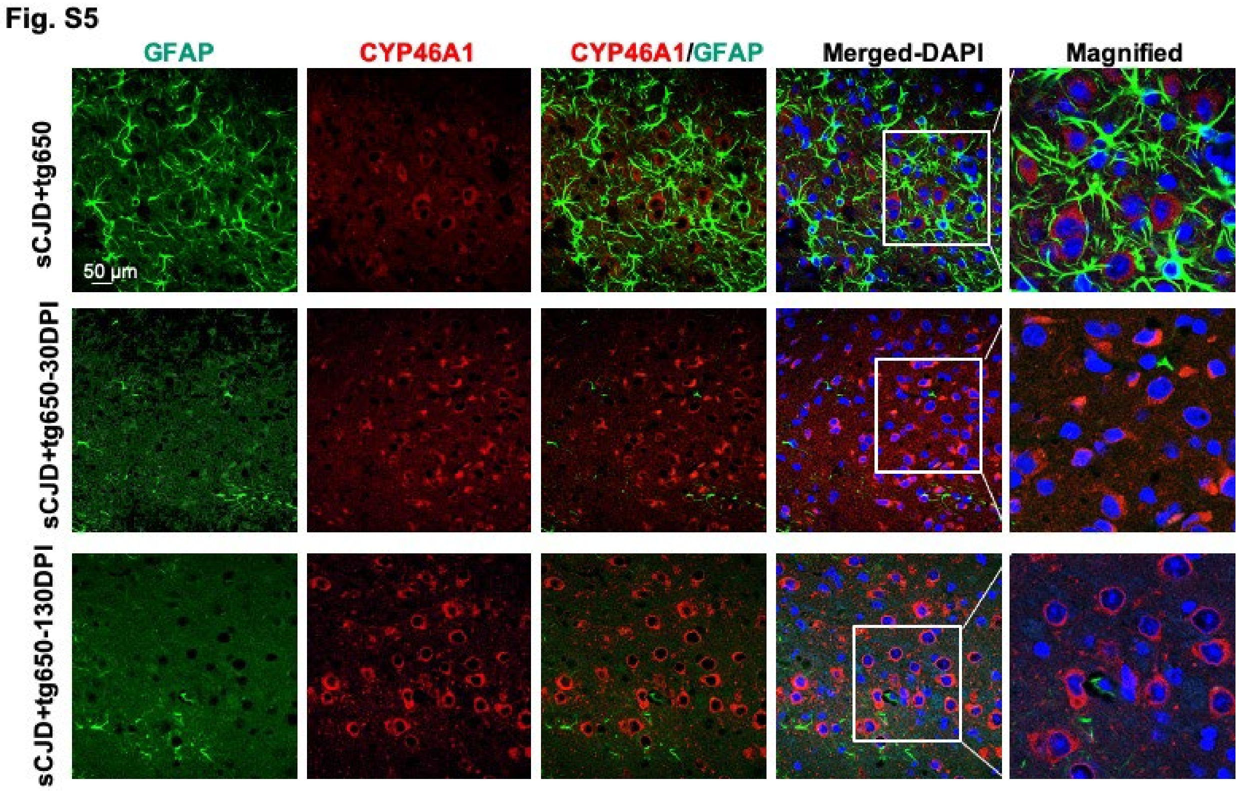

Fig. S5. Double immunofluorescence analysis of CYP46A1 and reactive astrocytes. Confocal images displaying double immunofluorescence staining for CYP46A1 (red) and GFAP (green) in the brains of sCJD-tg650, sCJD-tg650-30 DPI, and sCJD-tg650-130 DPI at pre-clinical stage (176 DPI). Magnification: 63X. Scale bar = 50 μm.

Fig. S6

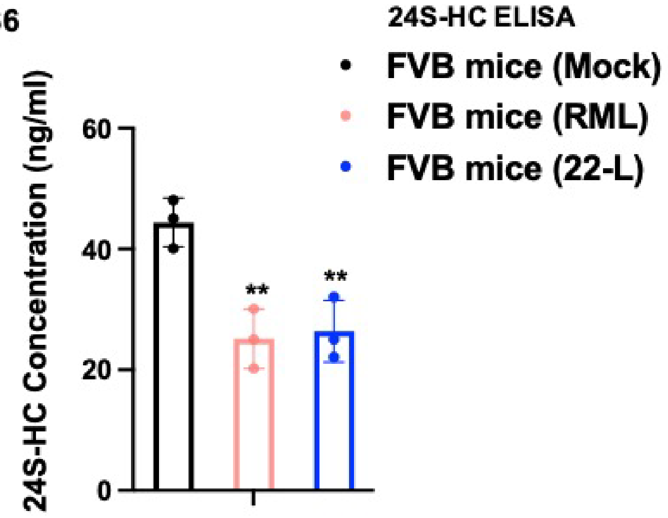

Fig. S6. ELISA quantification of the cholesterol metabolite 24S-hydroxycholesterol (24S-HC) in brain homogenates from FVB (non-infected, mock) mice and FVB mice infected with scrapie prions (RML and 22L). Histograms represent means ± SEM (n = 3 mice/group) from three independent experiments. Ordinary One-way ANOVA was performed, and statistical significance is $p<0.0041$.

Fig. S7

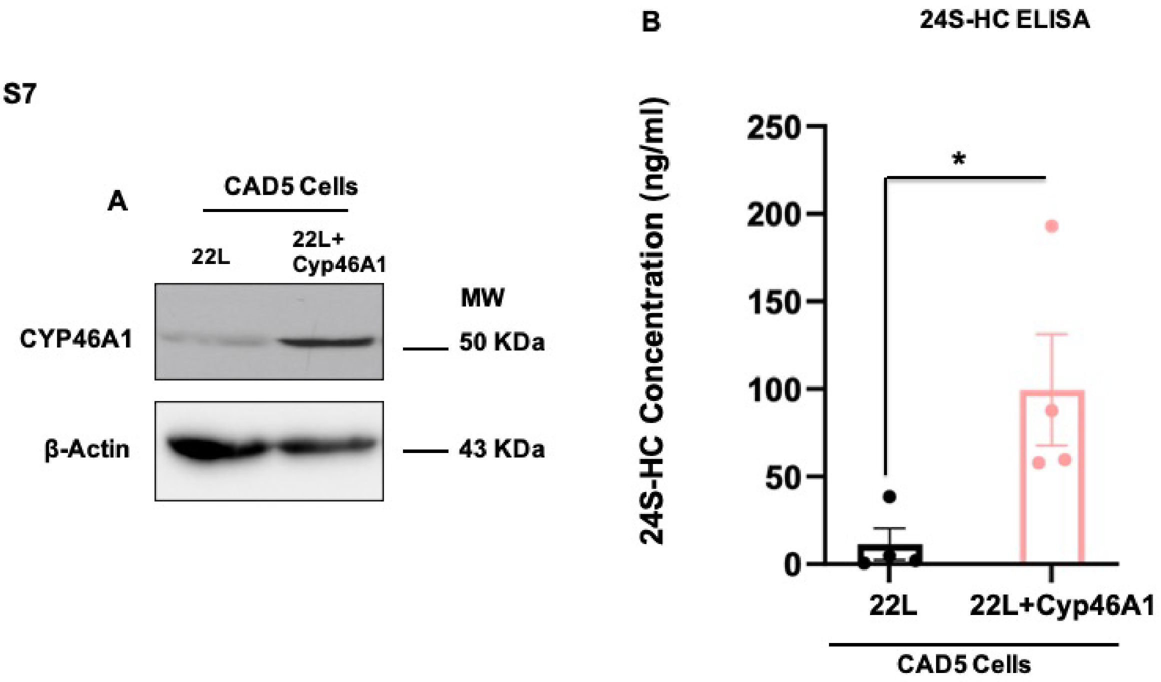

Fig. S7. (A) Immunoblot analysis of CYP46A1 in 22L-CAD cells and CYP46A1-overexpressing 22L-CAD5 cells. **(B)** ELISA quantification of the cholesterol metabolite 24S-hydroxycholesterol (24S-HC) in the media of 22L-CAD cells and CYP46A1-overexpressing 22L-CAD5 cells. The histograms represent the means ± SEM for n = 4 per group, obtained from 3 independent experiments. T-test (unpaired t test) was performed, and statistical significance is $p<0.05$.

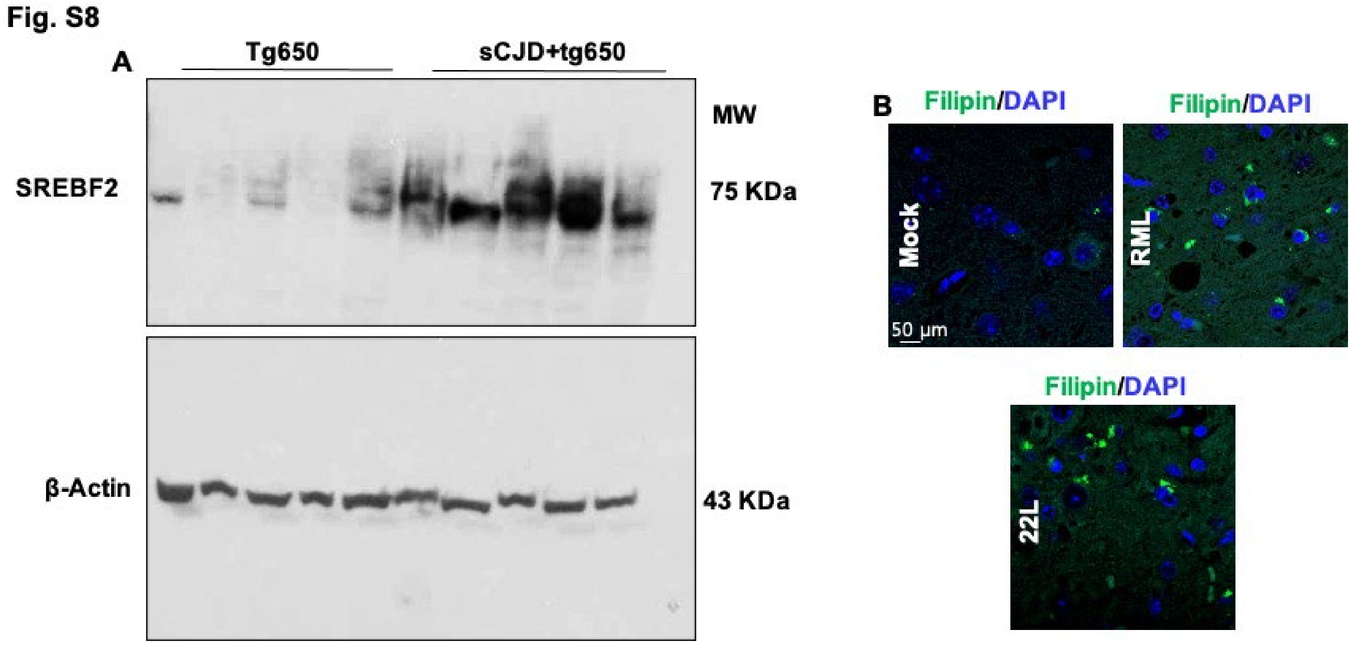

Fig. S8. (A) Analysis of uncropped immunoblot of SREBF2 protein in brain homogenates from five distinct non-infected (mock) tg650 mice and sCJD-tg650 mice at the terminal disease stage. **(B)** Filipin staining (green) and DAPI (blue) in brain sections from mock FVB mice and FVB mice infected with scrapie prions (RML and 22L). Magnification: 63X. Scale bar: 50 µm.
