# Supplementary material for: Treatment with efavirenz extends survival in a Creutzfeldt-Jakob disease model by regulating brain cholesterol metabolism
Source: JCI Insight. 2025 Jun 19;10(14):e190296. doi: 10.1172/jci.insight.190296 (PMC12288963; doi:10.1172/jci.insight.190296)

**PrP$^{Sc}$ uncropped WB for Fig. 2D**

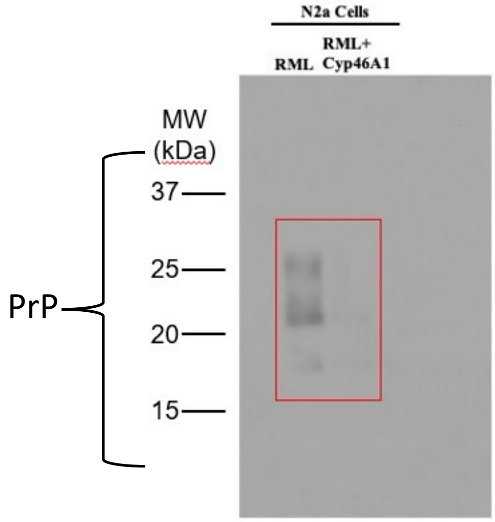


**PrP$^{Sc}$ uncropped WB for Fig. 2E**

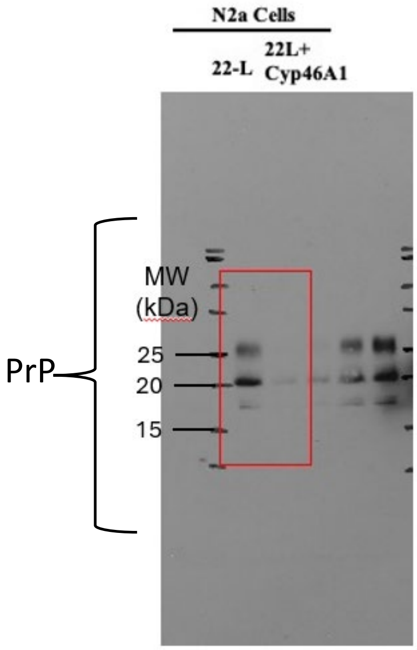

**CYP64A1 uncropped WB for Fig. 2D**

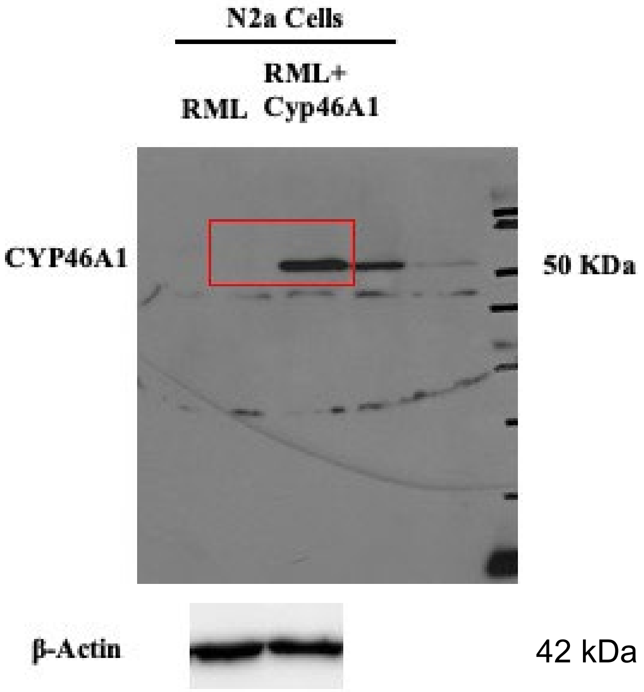


**CYP46A1 uncropped WB for Fig. 2E**

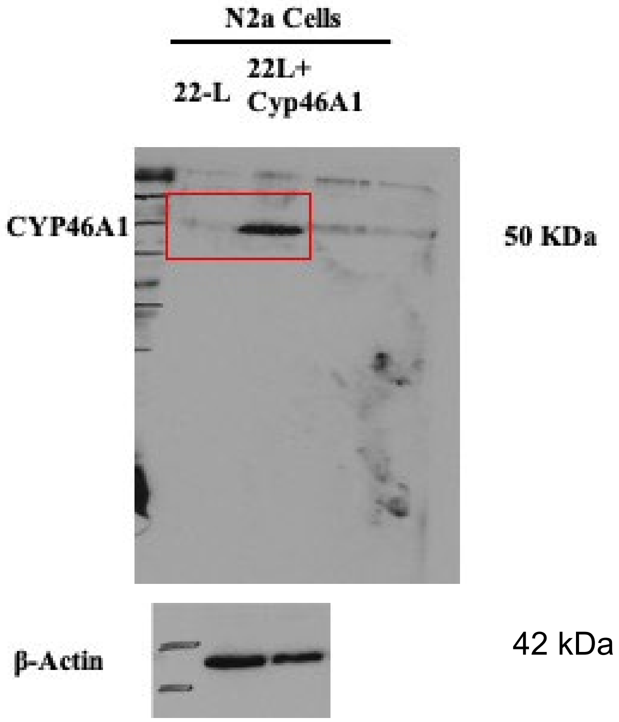


**Beta-actin of RML-N2a CYP46A1 blot**

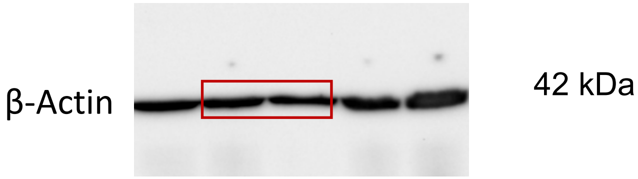

**Beta-actin of 22L-N2a CYP46A1 blot**

β-Actin

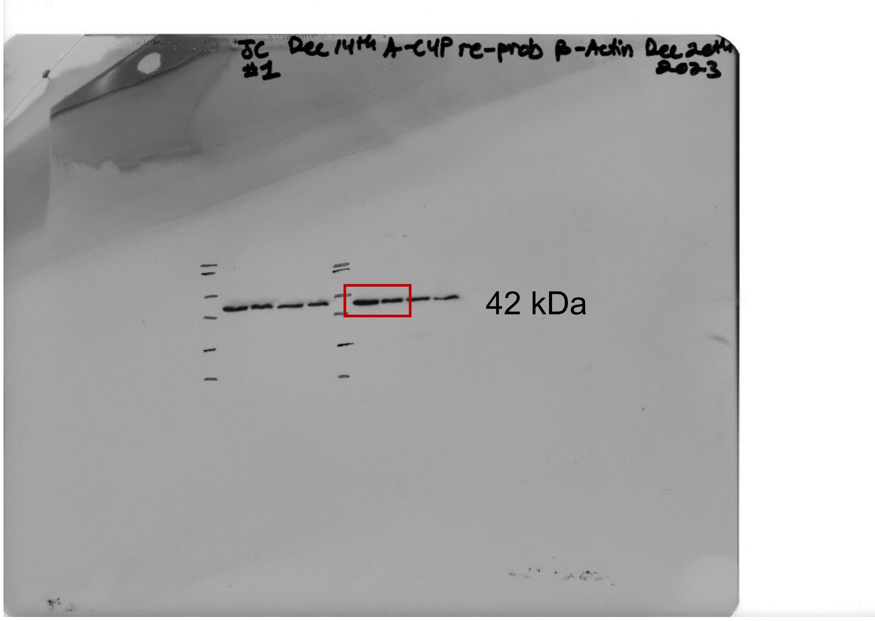


42 kDa

**CYP46A1 uncropped WB for Fig S7**

CYP46A1

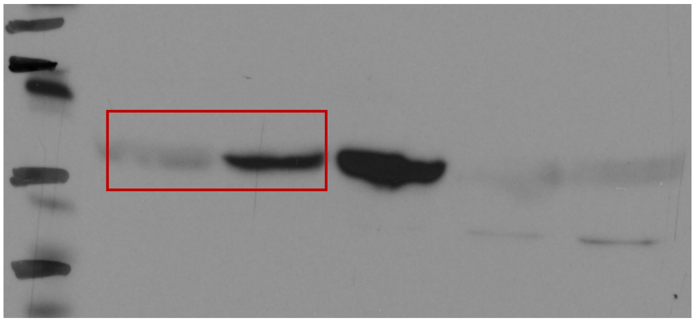

50 kDa

β-Actin

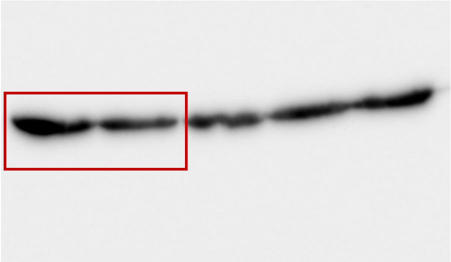

42 kDa

**Beta-Actin**

**CYP46A1 and beta-actin uncropped WB for Fig. 3A**

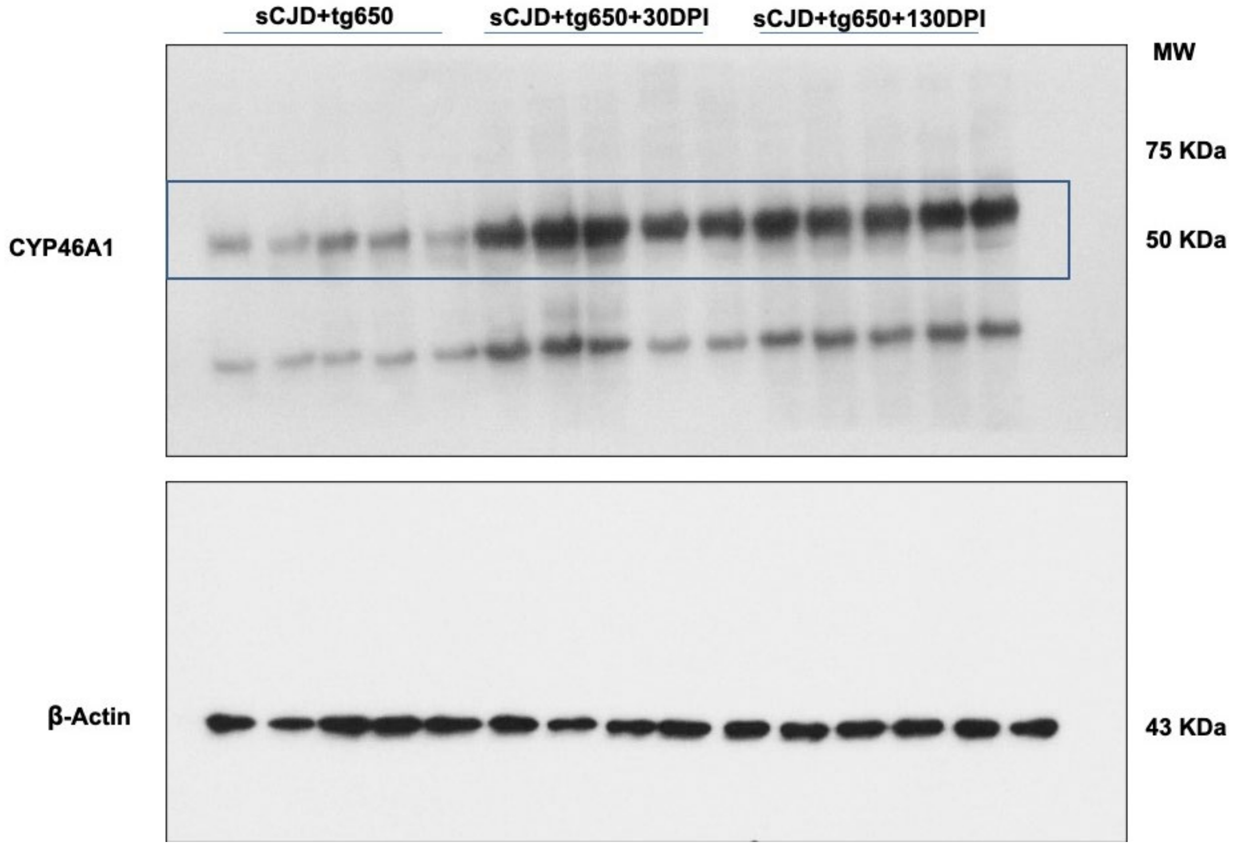

**SREBF1 and beta-actin uncropped WB for Fig. 4A**

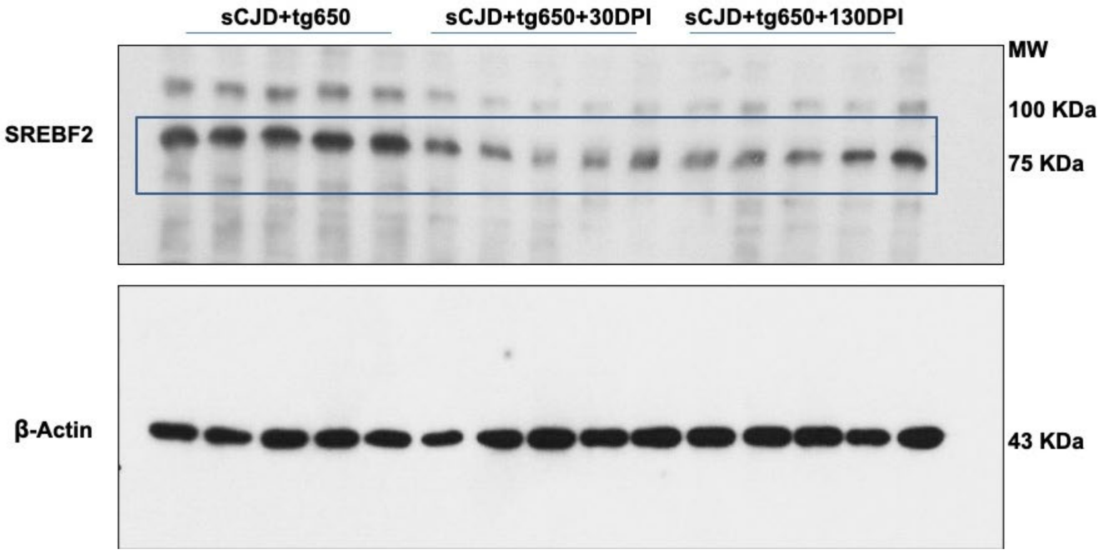

Supplement: Unedited blot and gel images [file jciinsight-10-190296-s052.pdf]
